# Nursing Home Surveyor and Survey Team Characteristics Across States

**DOI:** 10.1111/jgs.70201

**Published:** 2025-11-08

**Authors:** Robert J. Skinner, David G. Stevenson

**Affiliations:** ^1^ Department of Health Policy Vanderbilt University Medical Center Nashville Tennessee United States; ^2^ Geriatric Research Education and Clinical Center (GRECC), Department of Veterans Affairs Tennessee Valley Healthcare System Nashville Tennessee United States

**Keywords:** nursing homes, oversight, regulation, surveyors

## 1 Introduction

Since the 1987 Nursing Home Reform Act, nursing homes (NHs) are required to receive annual “recertification” surveys to continue participation in the Medicare and Medicaid programs and “complaint” surveys following allegations of poor‐quality care [1]. These onsite surveys review resident care quality and safety and are typically conducted by surveyors employed by state survey agencies (SSAs). Using unannounced visits [2], surveyors interview residents and staff, conduct records reviews, observe resident care, and conduct safety inspections [3]. In concluding the survey visit, surveyors identify any relevant deficiencies and may produce corrective action plans for NH staff to implement [3].

Prior studies have attempted to assess how NH surveys influence resident quality [4, 5]. For example, research has found that NHs respond to deficiency citations, improving care on dimensions of cited deficiencies, although these improvements may be offset by reduced NH focus on areas of care related to lesser cited deficiencies [5]. There is little evidence on how survey teams and surveyors may influence deficiency patterns, limiting insights of previous work that controls for state‐level trends and assumes SSA‐wide homogeneity in surveyor stringency. Additionally, the research literature has not categorized how surveyor team characteristics vary across states or how this variation affects survey quality. Although the Centers for Medicare & Medicaid Services (CMS) provides guidelines for the NH survey process in the State Operations Manual, SSAs are tasked with hiring surveyors and deploying survey teams [3, 6]. For example, federal regulations stipulate that surveys must contain at least one registered nurse (RN) on a recertification survey, but SSAs may vary the professional qualifications of the remaining survey team members [6, 7]. Our analysis assesses variation in the RN share of the survey workload. Although CASPER data include classifications for 36 possible surveyor professions, RNs predominate providing 66% of the surveyor‐survey workload (Table S1). Existing qualitative research suggests surveyor discretion can influence regulatory outcomes, but we are unaware of research exploring how surveyor team characteristics may influence these decisions [8]. Also, government reports have noted surveyor staffing challenges for decades [6, 9]. This paper augments these findings by assessing variation in surveyor staffing and workload across SSAs.

## 2 Methods

We examine NH surveyors and survey teams using a dataset of survey and surveyor‐level recertification and complaint surveys. We use Certification and Survey Provider Enhanced Reports (CASPER) data combined with surveyor‐level information from CMS for 2023 [10]. These 2023 data are used to produce state‐level descriptive statistics of the number of active surveyors (surveyors conducting at least one survey), surveyor team size, the number of NHs visited by individual surveyors, and RN involvement in NH surveys. To assess RN involvement, we calculate the proportion of surveyor‐survey combinations associated with RNs, summing the total number of RN surveyors on recertification and complaint surveys in 2023 for each state and dividing by the total number of surveyors. Additionally, we merge these data with information from Quality, Certification, and Oversight Reports from CMS for 2023 to acquire the number of NH residents in each state [11]. These data are used to estimate state‐level active surveyors per 1000 NH residents.

## 3 Results

**FIGURE 1:**
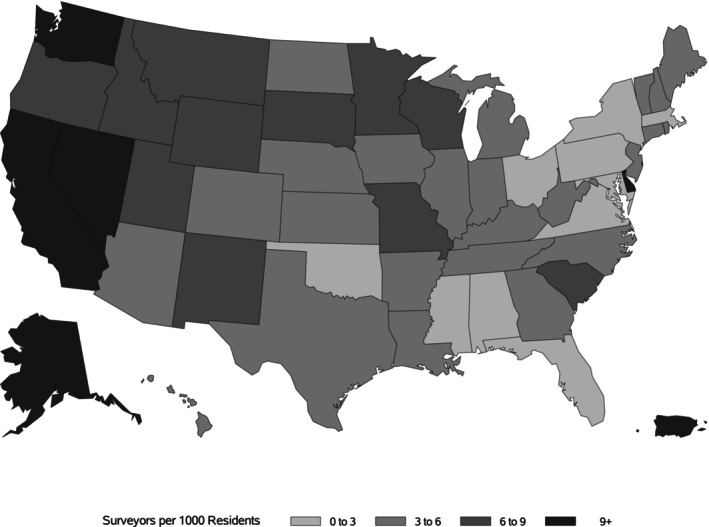
Number of active surveyors per 1000 nursing home residents, 2023. *Note:* Active Surveyors represent surveyors who performed at least one survey during 2023. *Source:* Number of active surveyors in 2023 is sourced from CMS surveyor staffing data. This number represents the unique count of surveyors who conducted any recertification or complaint survey in 2023. The number of residents in 2023 is sourced from the quality, certification, and oversight reports from CMS.

We find large variation in the number of active surveyors per 1000 NH residents across states. For example, we find that Alaska had 12.5 active surveyors per 1000 NH residents in 2023, compared to Alabama, Mississippi, New York, and Pennsylvania who had fewer than 2.5 surveyors per 1000 NH residents (Figure 1).

Similarly, we find variation in the number of surveyors per NH, ranging from 1 surveyor per NH in Puerto Rico to 0.14 surveyors per NH in Kansas. We find wide variation in the intensity of RN usage and in surveyor experience. In Alaska and Minnesota, 85% of surveyor‐survey combinations are associated with an RN compared to only 18% in Colorado and South Carolina. In Table 1, the number of NHs visited per surveyor and the proportion of surveyors with 4+ years of experience varies widely across states.

**TABLE 1:** Surveyor and survey team characteristics, 2023.

State	Number of nursing homes	Number of active surveyors	% registered nurses	Average number of surveyors per recertification survey	Average number of nursing homes visited per active surveyor	% surveyors with 4+ years of experience
National	15,075	5334	66%	2.9	18.6	45%
AK	20	9	85%	2.2	7.4	56%
AL	225	44	43%	2.8	7.7	30%
AR	226	53	82%	2.8	31.3	32%
AZ	143	62	24%	3.2	17.6	19%
CA	1173	903	90%	3.8	12.9	48%
CO	218	62	18%	2.7	17.7	39%
CT	203	65	84%	4.6	20.0	43%
DC	17	9	67%	3.0	9.6	44%
DE	44	35	76%	4.2	7.2	29%
FL	702	204	54%	3.1	23.2	56%
GA	358	172	51%	3.0	9.3	35%
HI	42	15	73%	2.7	14.3	53%
IA	416	115	76%	2.6	14.9	30%
ID	81	28	31%	3.3	5.6	29%
IL	696	236	86%	2.9	33.7	67%
IN	522	117	90%	2.6	26.4	62%
KS	312	45	83%	2.0	34.8	67%
KY	279	85	59%	3.9	5.3	18%
LA	269	131	94%	3.4	19.2	44%
MA	354	78	43%	3.4	25.4	53%
MD	225	50	70%	4.2	8.3	44%
ME	87	21	83%	2.8	18.1	57%
MI	430	101	57%	2.9	28.1	72%
MN	353	143	85%	2.4	18.8	32%
MO	512	265	48%	3.6	15.3	45%
MS	203	31	83%	2.6	31.7	42%
MT	62	23	49%	2.5	14.0	39%
NC	421	122	62%	2.8	18.6	55%
ND	77	21	72%	2.8	19.1	67%
NE	187	57	73%	2.4	14.8	25%
NH	74	19	49%	2.7	23.9	32%
NJ	349	172	63%	3.5	9.9	30%
NM	68	34	25%	2.8	11.3	38%
NV	66	52	63%	2.8	9.4	46%
NY	608	192	62%	3.6	16.7	43%
OH	947	146	62%	2.4	44.6	60%
OK	294	50	54%	2.1	29.2	50%
OR	129	41	35%	2.7	18.1	61%
PA	677	137	58%	2.5	36.1	64%
PR	6	6	61%	3.8	5.6	50%
RI	75	33	63%	3.7	30.0	30%
SC	190	137	18%	2.8	8.0	25%
SD	99	33	44%	2.6	14.4	33%
TN	312	98	76%	3.3	10.9	49%
TX	1208	452	41%	2.4	25.0	41%
UT	98	34	44%	2.8	11.9	21%
VA	290	71	75%	3.0	10.8	42%
VT	35	12	31%	3.4	19.7	42%
WA	197	138	69%	3.4	11.5	31%
WI	335	127	70%	2.7	19.2	35%
WV	125	36	50%	2.9	16.4	39%
WY	36	12	55%	2.0	12.6	58%
Mean	289.9	102.58	61%	2.9	17.8	43%
Interquartile Range	275	99.5	27%	0.7	12.5	22%

*Note:* The “% Registered Nurse” represents the proportion of surveyor‐survey combinations associated with Registered Nurse surveyors. These numbers represent the intensity of RN usage on surveys across states. The “Number of Nursing Homes Visited per Surveyor” includes complaint and recertification surveys in 2023. Active Surveyors represent surveyors that performed at least one recertification, complaint, or focused‐infection‐control survey during 2023. We note that a state that employs part‐time surveyors would have more active surveyors than states employing only full‐time surveyors. These differences would not necessarily reflect differences in SSA investment in oversight. The estimate of surveyor experience is constructed by estimating the proportion of surveyors active in 2023 who were also active in 2019 or earlier. These estimates of experience are limited to nursing home surveys. We are not able to estimate experience surveying other types of healthcare facilities or relevant experience outside of surveying (such as working as a nurse). The mean and inter‐quartile range are derived from the state‐level estimates.

*Source:* The number of providers comes from the Quality, Certification, and Oversight Reports from the Centers for Medicare and Medicaid Services. All other columns use Certification and Survey Provider Enhanced Reports Data and CMS Surveyor Staffing Information. All data represent calendar year 2023.

## 4 Discussion

Our results show wide variation in surveyor‐level metrics across SSAs. Existing government reports have categorized differences in surveyor vacancies and salaries across states [6, 9]. Our work adds further context, analyzing states' NH surveyor capacity and composition. Since the survey process involves interviewing residents and staff, the number of surveyors per resident may suggest a greater ability for SSAs to monitor NH quality. In addition, the greater usage of RN and experienced surveyors conveys resource investments that could bolster survey teams' effectiveness. The most common surveyor qualification after RNs is surveyor generalists, who have passed the surveyor qualification test and training course, but do not necessarily have other clinical expertise. We do not assess state variation in non‐RN surveyor qualifications. We provide the number of NHs visited per surveyor to show variation in the deployment of surveyors to NHs. Additionally, SSAs have reported challenges completing surveys post‐COVID‐19 due to staff turnover and a large survey backlog from the suspension of recertification surveys during the public health emergency [12]. Our 2023 findings overlap with this tumultuous period. In addition, our surveyor‐level metrics are not a proxy of SSA survey stringency which has been shown to vary across states [4]. Nonetheless, these surveyor‐level data provide insights into how states approach the NH survey process. Future work should explore the relationship between surveyor‐level metrics, survey stringency and performance, the timeliness of recertification and complaint surveys, and resident quality of care.

## Author Contributions

Robert J. Skinner and David G. Stevenson both engaged in the study concept and design, acquisition of data, analysis and interpretation of data, and critical revision of the manuscript. Robert J. Skinner drafted the manuscript.

## Conflicts of Interest

The authors declare no conflicts of interest.

## Supporting information


**Table S1:** Share of surveyor‐surveys by occupation in 2023.
