# Supplementary material for: Nursing Home Surveyor and Survey Team Characteristics Across States
Source: J Am Geriatr Soc. 2025 Nov 8;74(2):596–600. doi: 10.1111/jgs.70201 (PMC12911534; doi:10.1111/jgs.70201)
Supplement: Supplementary file 1 — Table S1: Share of surveyor‐surveys by occupation in 2023. [file JGS-74-596-s001.pdf]

Supplementary Table S1: Share of Surveyor-Surveys by Occupation in 2023

**CASPER Display Name Definition**	**Surveyor-Survey Share in 2023**	**Surveyor-Survey Counts in 2023**
registered nurse surveyors	65.94%	134028
generalist surveyors	15.85%	32211
social work surveyors	6.81%	13845
nutritional/dietary surveyors	3.74%	7608
other surveyors	3.26%	6625
Licensed Practical Nurse surveyors	0.94%	1916
sanitarian surveyors	0.84%	1700
pharmacist surveyors	0.46%	937
registered nurse surveyors who are also Qualified Mental Retardation Professionals	0.36%	732
occupational therapy surveyors	0.29%	597
life safety code specialist surveyors	0.28%	564
physical therapy surveyors	0.25%	513
nursing home administrator surveyors	0.22%	455
social work surveyors who are also Qualified Mental Retardation Professionals	0.16%	322
human service surveyors who are also Qualified Mental Retardation Professionals	0.12%	249
speech audiologist surveyors	0.10%	199
laboratorians or medical technicians	0.09%	179
recreational therapy surveyors	0.09%	177
nutritional/dietary surveyors who are also Qualified Mental Retardation Professionals.	0.06%	119
record administration surveyors	0.05%	103
recreational therapist surveyors who are also Qualified Mental Retardation Professionals	0.03%	64
health care surveyors	0.03%	56
medical doctor surveyors	0.02%	41
psychologist surveyors who are also Qualified Mental Retardation Professionals.	0.00%	6
psychologist surveyors	0.00%	5
occupational therapist surveyors who are also Qualified Mental Retardation Professionals	0.00%	4
physical therapist surveyors who are also Qualified Mental Retardation Professionals.	0.00%	4
engineering surveyors	0.00%	2
accredited administrator surveyors	0.00%	0
architect surveyors	0.00%	0

cytotechnologist surveyors	0.00%	0
doctor of osteopathy surveyors	0.00%	0
doctor of osteopathy surveyors who are also Qualified Mental Retardation Professionals	0.00%	0
doctor of medicine surveyors who are also Qualified Mental Retardation Professionals	0.00%	0
pathologist surveyors	0.00%	0
speech audiologist surveyors who are also Qualified Mental Retardation Professionals	0.00%	0

Note: All surveyors must complete the Basic Long Term Care Health Facility Surveyor Training Course and pass the Surveyor Minimum Qualifications Test, an exam which assesses the surveyor's ability to ensure compliance with Centers for Medicare & Medicaid Services standards. Surveyors are tested on topics such as the survey process, medicine, nursing, gerontology, nutrition, infection control, and skills in documenting information. We provide counts of the surveyor-survey share for the 36 occupations listed in the Certification and Survey Provider Enhanced Reports (CASPER) data. We use surveyor-survey share as our estimate of surveyor occupation intensity to measure not just the commonness of surveyors by profession but also the intensity of their use. We note that in CASPER 0.03% (56) of the surveyor-survey share is designated as "health care surveyors." CASPER does not provide documentation distinguishing a "health care surveyor" from other surveyor occupations.
